# Analysis of microsatellites from transcriptome sequences of *Phytophthora capsici* and applications for population studies

**DOI:** 10.1038/s41598-018-23438-8

**Published:** 2018-03-26

**Authors:** C. H. Parada-Rojas, L. M. Quesada-Ocampo

**Affiliations:** Department of Plant Pathology, North Carolina State University, Raleigh, NC 27695 USA

## Abstract

*Phytophthora capsici* is a devastating oomycete that affects solanaceous, cucurbitaceous, fabaceous, and other crops in the United States (US) and worldwide. The release of the *P. capsici* genome allows for design of robust markers for genetic studies. We identified and characterized microsatellites in the *P. capsici* transcriptome. A subset of 50 microsatellites were assayed in a diverse set of *P. capsici* isolates and evaluated for polymorphism. Polymorphic microsatellites were confirmed by fragment analysis, and 12 were used for population characterization of 50 *P. capsici* isolates from different states, hosts, and mating types. Analysis of genetic relationship among isolates revealed significant geographic structure by state. Our findings highlight the usefulness of these 12 microsatellites to characterize the population structure of *P. capsici* and potential transferability to closely-related *Phytophthora* spp. since markers are located in coding regions. Our markers will facilitate genetic characterization and complement phenotypic studies of *P. capsici* populations, which may assist in deployment of disease management strategies.

## Introduction

The oomycete *Phytophthora capsici* Leonian is a devastating hemibiotrophic pathogen that causes severe epidemics over a broad host range of crops, including peppers, cucurbits, tomatoes, snap beans, eggplants, and many other plant species in the United States (US) and worldwide^1–6^. *P. capsici* can produce numerous symptoms depending on the host, including foliar blighting, damping-off, wilting, and root, stem, and fruit rot^3,4^. In the field, disease management relies primarily on the application of fungicides, in combination with cultural practices that are unfavorable for disease development such as raised beds, plastic mulch, and drip irrigation^3^. However, the broad host range of *P. capsici* and its capability to undergo sexual recombination and produce thick-walled oospores as persistent survival structures^7^ has limited the efficacy of control strategies such as crop rotation^8,9^. Sexual recombination between the A1 and A2 mating types of this heterothallic oomycete can result in novel genotypes with increased virulence, pathogenicity, and the ability to overcome host resistance and fungicides^3^. *P. capsici* sporangia are infrequently dispersed among fields by wind^10^. Instead, surface water sources for irrigation and movement of infected plant material or infested soil are known to be key factors in local spread of *P. capsici* and in the development of epidemics^11^. Because of limited long-distance dispersal of *P. capsici* and its ability to quickly overcome resistant varieties and fungicides due to frequent sexual reproduction, a better understanding of regional pathogen population structure would allow for more effective deployment of resistant varieties and breeding for durable host resistance^12^.

The amount and distribution of genetic diversity in *P. capsici* populations have been studied at a global scale within countries^8,12–15^ and at a local scale within states in the US^10,16–19^. Studies employed a wide range of molecular markers such as random amplified polymorphic DNA (RAPD)^15,19,20^, amplified fragment length polymorphism (AFLP)^10,21,22^, and single nucleotide polymorphism (SNP)^8,13^. Nonetheless, despite the benefits offered by microsatellites for population analyses^23^, only a few useful microsatellite loci have been developed to assess genetic diversity on *P. capsici* population studies^14,18^. Microsatellites, also known as simple sequence repeats (SSRs), are short DNA sequences consisting of tandemly repeated units, generally 1–6 base pairs in length^24,25^. Microsatellites can be found in either non-coding or coding regions and are highly polymorphic, making them desirable for population genetic analyses^26,27^. Microsatellites have been widely used in recent studies as a marker for diagnostics, population structure assessments, and epidemiological studies in oomycetes^28–33^. Genetic diversity in *P. capsici* has also been determined by microsatellites^14,16,18,34,35^, however, most of these studies relied on a set of microsatellites designed from a database of expressed sequence tags (ESTs)^14,18^. To date no microsatellite markers have been developed from the current *P. capsici* genome assembly, which could offer a set of standardized markers to be used by the scientific community^35^.

Traditionally, the methodology for microsatellite mining involved enrichment of genomic DNA libraries for a few targeted microsatellite motifs, followed by screening and sequencing of clones through Sanger sequencing. The entire process is labor intensive, resource consuming, and usually yields a small number of polymorphic markers^36,37^. The publication of the *P. capsici* genome, predicted transcriptome^38^, and the availability of microsatellite search tools, such as MISA (MIcroSAtellite identification tool)^39^, allow mining for microsatellites from whole genomes or transcriptomes. Identifying microsatellites in transcript sequences implies the identification of polymorphisms within coding regions, which represent annotated markers located in genes that may play important roles in pathogen virulence and survival^40^. This approach has been successfully applied to identify microsatellites in an increasing number of species, including *Phytophthora* species such as *P. nicotianae*^28^, *P. ramorum*^41,42^, *P. sojae*^41,42^, *P. infestans*^42^, *P. plurivora*^32^*, P. multivora*^32^*, P. pini*^32^, and plant pathogens such as *Alternaria brassicicola*^43^, *Gaeumannomyces graminis*^44^, *Erysiphe necator*^45^, *Fusarium verticillioides*^46^, *F. oxysporum*^47^, *Anisogramma anomala*^48^, and more recently *Synchytrium endobioticum*^49^. To date, development of microsatellite markers from transcript sequences in *P. capsici* has been unreported, despite the advantage of such studies and the economic importance of the pathogen. We aimed to: (i) identify and characterize microsatellites from the publicly available transcriptome of *P. capsici* using an *in silico* approach; (ii) determine microsatellite polymorphism in a diverse selection of *P. capsici* isolates and evaluate them in a population study of 50 isolates; and (iii) compare the distribution of microsatellites in the *P. capsici* transcriptome with the transcriptomes of two other plant pathogenic oomycetes (*P. sojae* and *P. ramorum*). The characterized polymorphic microsatellites in this study will help in understanding regional pathogen population structure, which will result in improved disease management strategies.

## Results

### Microsatellite identification and analysis

The *in silico* search for microsatellites using MISA (MIcroSAtellite identification tool) examined 20.36 Mb of the transcriptome of *P. capsici* (v. 1.1). 8.17% of the sequences contained microsatellites (Supplementary Table S3). A total of 1,855 microsatellites were identified and among those 75 were found more than once in a single gene. Trinucleotide repeats represented the most prevalent motif length in transcript sequences across the species (Table 2). Among the different types of repeats in *P. capsici*, trinucleotide repeats were the most common, accounting for 71.00% of all repeats, followed by tetranucleotides (13.98%), dinucleotides (9.33%), hexanucleotides (4.53%), and pentanucleiotides (1.62%). The *P. capsici* transcriptome is smaller than *P. ramorum*, and *P. sojae*, and it contains less microsatellites. The amount of microsatellites among these *Phytophthora* species seems to be highly correlated with their genome size (Pearson, r = 0.989, *P* > 0.05 true correlation is not equal to 0). When accounting for the differences in length of examined sequences between species, the *P. capsici* transcriptome exhibited the lowest relative abundance (microsatellite/Mb) and relative density (bp/Mb) (91.09 microsatellite/Mb and 1280.05 bp/Mb respectively), compared to *P. ramorum* (145.75 microsatellite/Mb, 2181.37 bp/Mb, *P* < 0.05) and *P. sojae* (196.04 microsatellite/Mb, 3190.98 bp/Mb, *P* < 0.05) (Supplementary Table S3). Overall, the *P. capsici* transcriptome consists of significantly fewer microsatellites compared to the other *Phytophthora* species.

The frequency, relative abundance, and relative density of each repeat unit in the *P. capsici, P. ramorum*, and *P. sojae* transcriptomes is presented in Table 2. Results indicated high similarity among the frequencies, relative abundances, and densities of the same repeat unit across the three species. For all three transcriptomes, the trinucleotide microsatellites were the most common motif length corresponding to around 70.00% of the microsatellites. *P. capsici* has higher frequency, relative abundance and density of tetranucleotide microsatellites when compared to *P. ramorum*. *P. sojae* has the highest relative abundance and density of all microsatellite motifs when compared to *P. capsici* and *P. ramorum*.

The frequency of the most abundant microsatellite motifs in the three *Phytophthora* species is shown in supplementary figure S1, each species harbored a predominant set of microsatellite motifs. The primary dinucleotide motif in *P. capsici* was AC/GT whereas CG/CG was more common for *P. ramorum* and *P. sojae*. The main trinucleotide motif included AAG/CTT for *P. capsici* and AGC/CTG for *P. ramorum* and *P. sojae*. In concordance with dinucleotide and tetranucleotide motifs frequencies, *P. capsici* presents a different primary tetranucleotide motif (ACGG/CCGT) when compared with *P. ramorum* and *P. sojae* (AGCG/CGCT). Pentanucleotide motifs and hexanucleotides motifs showed the lowest frequencies (<1%) across the three species. In all species, regardless the motif, the microsatellites tended to repeat 3, 4, and 5 times more frequently, and very few microsatellites repeat >10 times (Supplementary Fig. 2).

**Figure 1:**
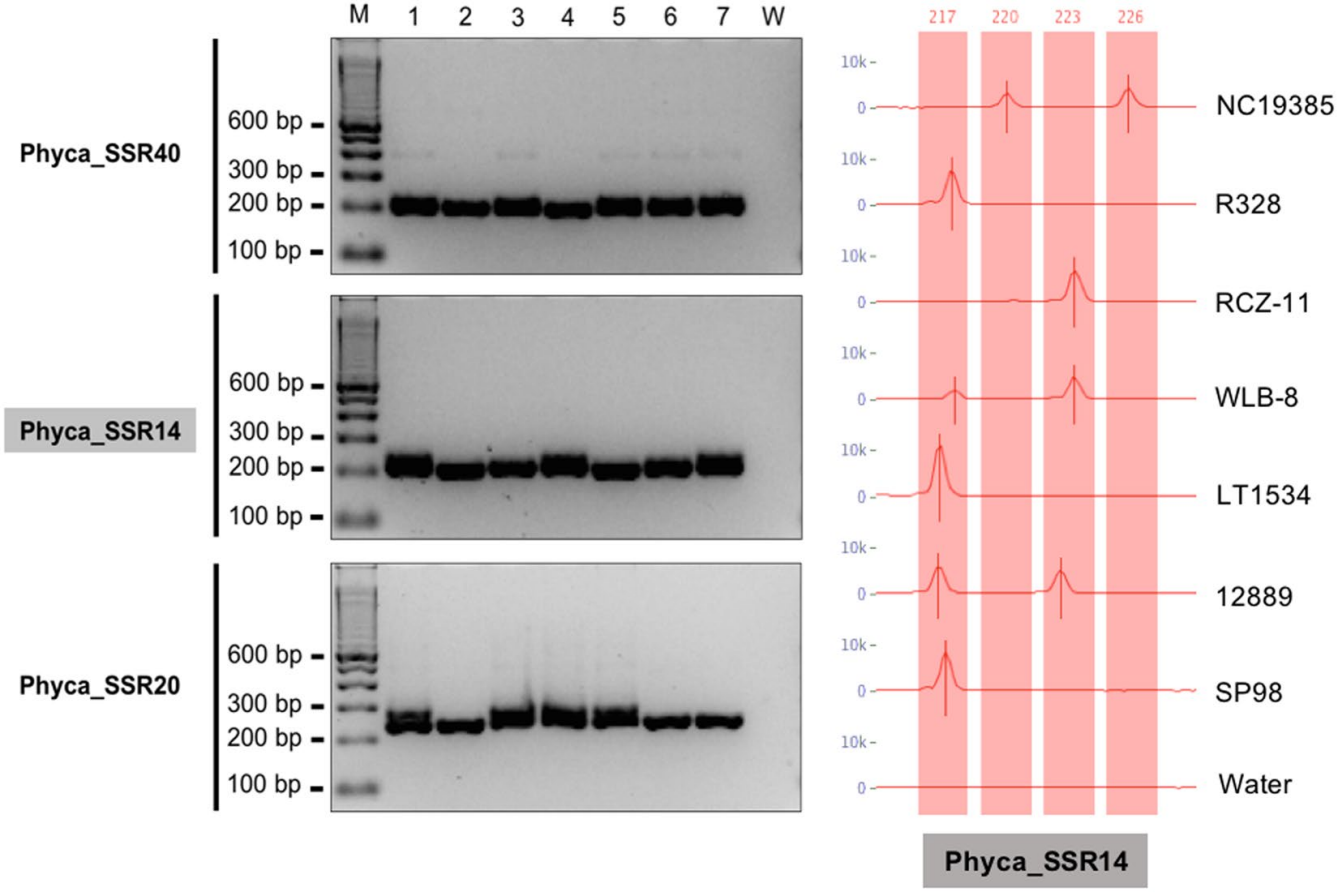
Amplification profile from 7 *P*. capsici isolates using the Phyca_SSR40, Phyca_SSR14, and Phyca_SSR20 primers. M: 100 bp marker, 1: NC19385, 2: R328, 3: RCZ-11, 4: WLB-8, 5: LT1534, 6: 12889, 7: SP98, and W: water. PCR products were resolved in 4% agarose gel. Fragment analysis profile for Phyca_SSR14 across the 7 *P. capsici* isolates, ordered as in the gel. The gels are cropped from different parts of different gels. Full-length gels are included in supplementary Figs S6, S7, and S8.

Primer3 successfully designed 1,491 primers pairs (80.37%) to flank microsatellites in the *P. capsici* transcriptome (Supplementary Table S4). Of the primers designed, 147, 1,015, 201, 21, and 59 belong to each of the motif lengths di-, tri-, tetra-, penta-, and hexa-nucleotide respectively. We randomly selected and synthesized 50 microsatellite primer pairs from 5 different motif lengths for agarose gel evaluation (Supplementary Table S2). Forty eight primers pairs (96.00%) successfully amplified and produced clear bands on 4% agarose gels. Thirty four (75.00%) out of 48 primer pairs yielded amplicons at the expected size. In total, 17 microsatellite primers exhibited polymorphism among the seven *P. capsici* isolates tested. Trinucleotide repeats yielded the highest number of polymorphic amplicons with nine primers exhibiting consistent polymorphic bands. Di-, Tetra-, Penta-, and Hexanucleotide primer pairs exhibited the lowest number of polymorphic bands. Figure 1 exemplifies allelic variation for Phyca_SSR14, Phyca_SSR20, and Phyca_SSR40 across 7 *P. capsici* isolates in 4% agarose gels. The fragment analysis profile for Phyca_SSR14 is presented for all tested isolates.

### Population analysis with microsatellites

Among the 17 microsatellites considered polymorphic in the agarose gels, we selected the 12 top performing based on consistent amplification and clear polymorphism in their agarose profile for further examination via fragment analysis in a broader panel of isolates. The microsatellite primers selected were used to assess the genetic relationship of 50 *P. capsici* isolates from different states in the US, exhibiting both mating types, and collected from different hosts (Table 1).

**Table 1:** 50 *Phytophthora* c*apsici* isolates used for microsatellite evaluation via fragment analysis.

Isolate	State^a^	County	Host	Mating Type	Year collected
GACP68	GA	Rabun	Pumpkin	A1	2016
NC16–025	GA	Rabun	Pumpkin	A2	2016
NC16–026	GA	Rabun	Pumpkin	A2	2016
NC16–027	GA	Rabun	Pumpkin	A2	2016
NC16–028	GA	Rabun	Pumpkin	A1	2016
12889	MI	UK^b^	Pepper	A1	1998
SP98	MI	UK	Pumpkin	A2	1998
NC16–024	NC	McDowell	Pepper	A2	2015
NCCP18	NC	Wilson	Creek	A2	2015
NCCP20	NC	Wilson	Creek	A1	2015
NCCP24	NC	Guilford	Zucchini	A2	2015
NCCP25	NC	Guilford	Zucchini	A2	2015
NCCP29	NC	Guilford	Zucchini	A1	2015
NCCP33	NC	Wilson	Watermelon	A2	2015
NCCP36	NC	Wilson	Cucumber	A1	2015
NCCP37	NC	Wilson	Cucumber	A2	2015
NCCP38	NC	Wilson	Cucumber	A2	2015
NCCP42	NC	Wilson	Cucumber	A1	2015
NCCP43	NC	Wilson	Cucumber	A2	2015
NCCP44	NC	Wilson	Cucumber	A1	2015
NCCP47	NC	Wilson	Cucumber	A1	2015
NCCP50	NC	Wilson	Cucumber	A2	2015
NCCP53	NC	Wilson	Cucumber	A1	2015
NCCP54	NC	Wilson	Cucumber	A1	2015
NCCP56	NC	Wilson	Cucumber	A1	2015
NCCP60	NC	McDowell	Snap Beans	A1	2015
R2	NC	Sampson	Pepper	A1	1989
R293	NC	Sampson	Pepper	A1	1989
R380	NC	Sampson	Pepper	A2	1989
6180	NY	Ontario	Winter Squash	A2	2006
0664-1	NY	Monroe	Pepper	A1	2006
EH-21A	NY	Ontario	Pumpkin	A1	2013
EH-84A	NY	Ontario	Pumpkin	A2	2013
MM2-6A	NY	Suffolk	Pepper	A2	2007
MML-03A	NY	Suffolk	Pumpkin	A2	2007
MMZ-46C	NY	Suffolk	Pumpkin	A1	2007
LC4	SC	UK	Sponge Gourd	A2	2014
MC1	SC	UK	Bitter Gourd	A2	2014
MC2	SC	UK	Bitter Gourd	A2	2014
RCZ-11	SC	Bamberg	Zucchini	A2	2003
WLB-150	SC	Beaufort	Watermelon	A2	2011
WLB-231	SC	Beaufort	Watermelon	A1	2011
WLB-232	SC	Beaufort	Watermelon	A1	2011
LT249	TN	Grainger	Pumpkin	A2	2004
LT251	TN	Grainger	Pumpkin	A1	2004
LT261	TN	Grainger	Pumpkin	A1	2004
LT262	TN	Grainger	Pumpkin	A1	2004
LT263N	TN	Grainger	Pumpkin	A2	2004
LT267	TN	Grainger	Pumpkin	A2	2004
LT268	TN	Grainger	Pumpkin	A1	2004

^a^States: NC: North Carolina, SC: South Carolina, TN: Tennessee, NY: New York, MI: Michigan, and GA: Georgia. Isolates were obtained from collaborators Dr. C. Smart, Dr. S. Kousik, Dr. M. Hausbeck, Dr. K. Lamour, and Dr. J. Ristaino.

^b^Unknown county of origin.

**Table 2:** Frequency, relative abundance, and relative density of each motif length in *P. capsici*, *P. ramorum*, and *P. sojae* transcriptomes.

	Pathogen	Frequency (%)^a^	Relative abundance^b^	Relative density^c^
Di-	*P. capsici*	9.33	8.50	85.54
	*P. ramorum*	12.20	17.79	167.27
	*P. sojae*	13.38	26.24	246.12
Tri-	*P. capsici*	71.00	64.67	754.71
	*P. ramorum*	73.64	107.34	1218.81
	*P. sojae*	69.55	136.34	1508.64
Tetra-	*P. capsici*	13.48	12.28	144.57
	*P. ramorum*	6.41	9.34	108.49
	*P. sojae*	8.85	17.35	194.61
Penta-	*P. capsici*	1.62	1.47	21.36
	*P. ramorum*	0.76	1.11	16.01
	*P. sojae*	1.71	3.34	44.58
Hexa-	*P. capsici*	4.53	4.12	62.76
	*P. ramorum*	6.96	10.14	178.75
	*P. sojae*	6.50	12.74	201.93

^a^Frequency was calculated for each organism by dividing the total number of each motif length by the total number of microsatellites count.

^b^Relative abundance is defined as number of microsatellites of each motif length per Mb of analyzed sequence.

^c^Relative density is defined as the total length of microsatellites (bp) per Mb of analyzed sequence.

The number of alleles per locus in the screened panel of isolates were, on average, 3.42 and ranged from 2 to 6 alleles. Heterozygosity varied from 0.38 to 0.79, with an average of 0.55. The evenness of alleles at each locus ranged from 0.63 to 1.0, with the mean evenness of 0.83 (Table 3). The panel of 50 *P. capsici* isolates were distributed across 50 multilocus genotypes. The genotype accumulation curve established 11 microsatellite markers as the minimum number of loci necessary to discriminate between the 50 *P. capsici* isolates (Supplementary Fig. S3). After clone correction, the standardized index of association (${\bar{r}}_{d}$) for all isolates (N = 50) was 0.019, which falls outside of the distribution expected under no linkage. The *p* value of 0.002 indicates significant support for rejecting the hypothesis that alleles are unlinked across loci, suggesting that the isolates used in this study are not recombining (Supplementary Fig. S4). Shannon-Wiener’s index was 3.912 indicating high genetic diversity in the panel of *P. capsici* isolates (N = 50). NC isolates showed the highest genetic diversity with Shannon-Weiner’s index of 3.091.

**Table 3:** Genetic diversity statistics for 12 microsatellite loci confirmed in 50 *P. capsici* isolates.

SSR id	Gene id	SSR motif	Primer sequence	Allelic size range	Na	He	Evenness
Phyca_SSR07	527040	(GA)6	F: CTCTGGCATTGAAAGAGCGC R: CCCAAAGTTGCGCCATTTGA	352–358	3	0.62	0.90
Phyca_SSR11^z^	101366	(CAG)4	F:CAGCAACAGCAACAGTCGTC R:TCCAAGTCGCTCGTCTGAAC	178–223	5	0.68	0.79
Phyca_SSR13	13824	(CAG)5	F:GAACACATCCGATTCGCAGC R:TTGCTGCTCAGATCCACTGG	122–134	4	0.65	0.78
Phyca_SSR14	7601	(AAG)6	F:CAGAAACACACGTCTCCGGA R:GTTCGAACTGCTCCTGCTCT	217–229	5	0.50	0.64
Phyca_SSR15	129784	(AGC)4	F:TCGTCGTTTTCCTCTGTGCA R:TTGAACTTCATCGCAGCCCT	178–181	2	0.45	0.90
Phyca_SSR17	15345	(AAG)6	F:TATCGGACGTTCTCGCCATG R:TGAGCGGTTTCTGCTCGAAT	126–129	2	0.50	1.00
Phyca_SSR18	121654	(AGC)6	F:GGACGATATCATGCAGCCGA R:CCGAGTCTGAACCCGAAGAG	271–280	3	0.47	0.82
Phyca_SSR20	103897	(AAG)7	F:CACGGAAGCTCAACGCAAAA R:GAGGTTGTCAGTGCTGTCGA	246–258	4	0.58	0.75
Phyca_SSR30	528924	(CCAG)6	F:CACAGCCTCTCGACCGGA R:CGTTTTCCAGCACACCCTTG	286–306	5	0.79	0.91
Phyca_SSR40	572218	(TCCTC)3	F:CAAGTCCCTGTCGTCGTTCT R:CATGGCAGTCACCGTCTCTT	210–215	2	0.47	0.94
Phyca_SSR41	570597	(CACGAC)5	F:GACTACGACGTCTACCGCTG R:GACGTCGTGGTGGTCGTAG	105–123	3	0.38	0.63
Phyca_SSR50	97293	(ACTTCA)4	F:GGGGCAGAAACGTCTCTGAA R:GGTCGTCGTCTGAGTCTGAC	237–249	2	0.48	0.95
Mean					3.42	0.55	0.83

Na: Number of alleles.

He: Nei (1978) gene diversity.

Evenness is a measure of the distribution of Multilocus Genotypes (MLGs) within the isolates (Pielou, 1975; Grünwald *et al*.^71^).

**Figure 2:**
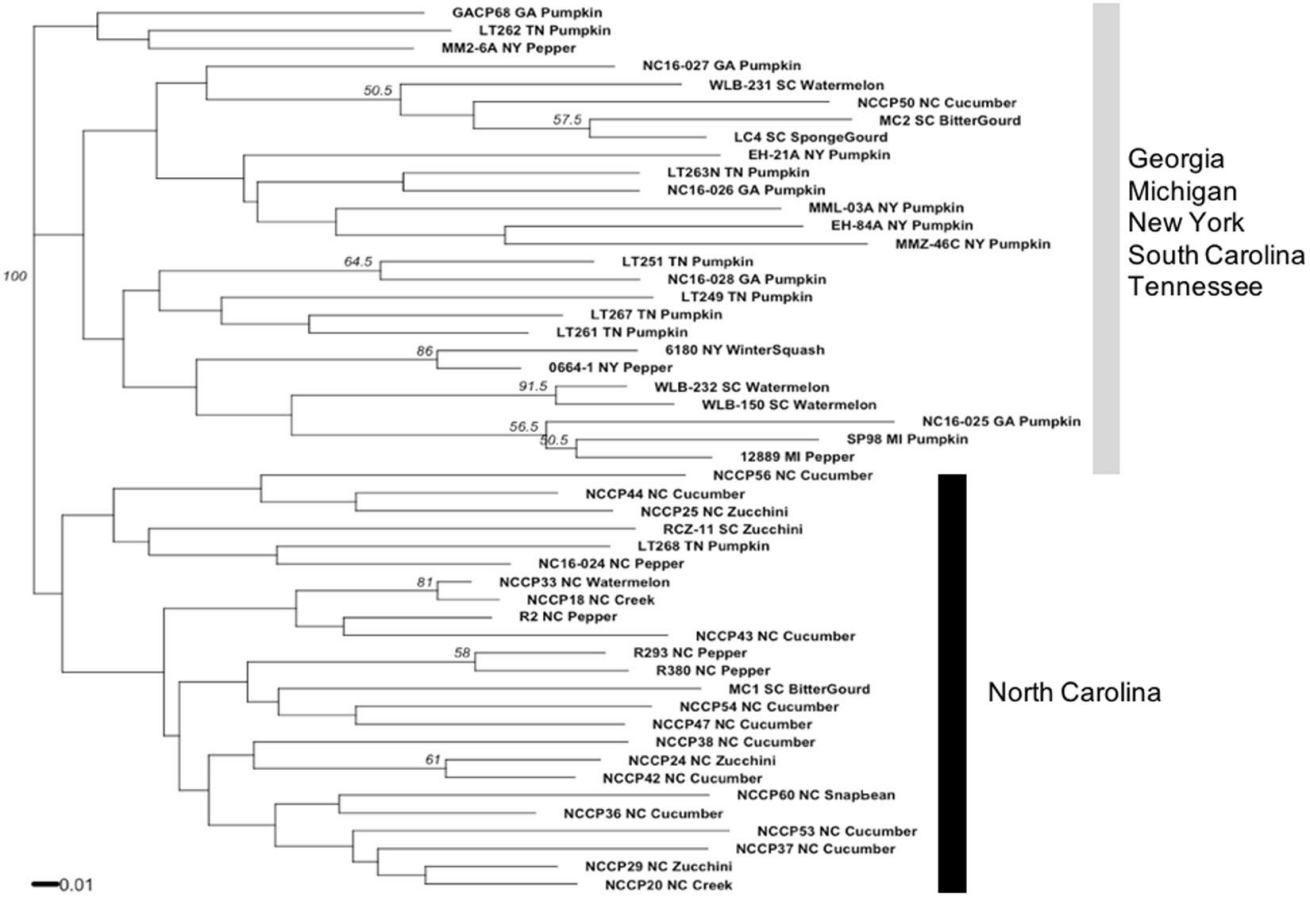
UPGMA tree with 1,000 bootstrap replicates based on Provesti’s distance for 50 *P*. capsici isolates. Tip labels define the isolate information by isolate name, state (NC: North Carolina, SC: South Carolina, NY: New York, GA: Georgia, TN: Tennessee, and MI: Michigan), and host. Branches with bootstrap values greater than 50% are displayed.

**Figure 3:**
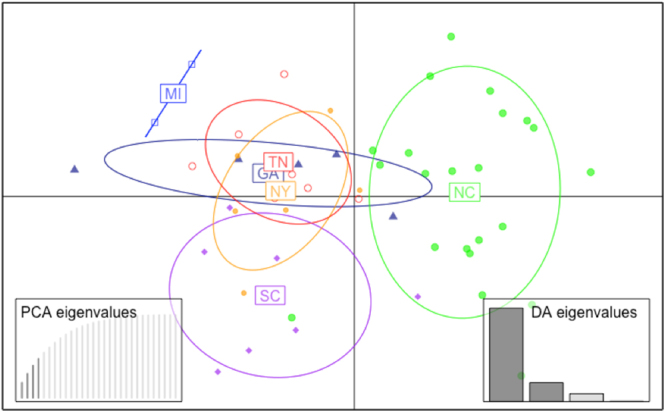
DAPC scatterplot of 50 *P*. *capsici* isolates sampled from 6 different states. Points represent individual isolates and different colors represent different states.

**Figure 4:**
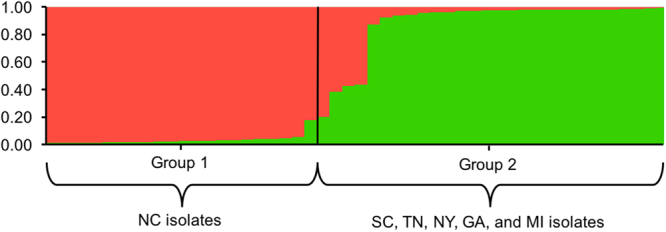
Population structure histogram for 50 *P. capsici* isolates when analyzed with 12 microsatellite markers using STRUCTURE. Color on each bar indicates membership probability of belonging to a corresponding group.

Analysis of genetic relationship among isolates using Provesti’s distance exhibited significant bootstrap support to separate the main group of isolates from North Carolina, which were collected from different fields across the state (Fig. 2). The other group comprised isolates from SC, TN, NY, GA, and MI. The genetic distance tree grouped some SC (WLB-232 and WLB-150) and NY (6180 and 0664-1) isolates closely with significant bootstrap support (>70%). Overall, the genetic distance tree separated isolates into two clusters, one with mainly NC isolates and the other with isolates from other states (Fig. 2). Population differentiation was revealed using pre-defined populations by states in the Discriminant Analysis of Principal Components (DAPC) (Fig. 3). DAPC is a multivariate statistical approach that partitions variance in the sample into a between-group and within-group component, in an effort to maximize discrimination between groups. *P. capsici* isolates from TN, NY, GA, SC, and MI overlapped in the first principal component, while NC isolates clustered separately from the other isolates. TN, NY, GA, and MI separated from SC in the second principal component (Fig. 3). The separation of NC isolates in the DAPC analysis supports the grouping observed in the Provesti’s genetic distance tree. Results from Analysis of Molecular Variance (AMOVA) revealed significant levels of differentiation among states and host (*P* < 0.005). Across states, nearly 13% of variation was attributed to differences among states, while more than 16% was attributed to variation between isolates within states, and 69% was attributed to variation within isolates. Only 6.38% of variation between isolates was attributed to differences due to host. Pairwise comparison (F_ST_) between states also showed significant differentiation between *P. capsici* isolates from NC and all other states included in this study (Table 4). *P. capsici* isolates from TN, NY, and GA exhibited low levels of genetic differentiation between themselves, which confirms overlap observed in the DAPC analysis. MI isolates showed moderate and high levels of differentiation when compared with NC, SC, and NY (*P* < 0.005) (Table 4). The STRUCTURE analysis based on the Bayesian clustering method established the presence of two population clusters, Group 1 and Group 2 (Fig. 4). The delta K plot indicated a clear peak at K = 2 (Supplementary Fig. S5). Twenty one *P. capsici* isolates included here from NC fields were assigned to Group 1 with membership probabilities higher than 0.82 (Fig. 4). *P. capsici* isolates from TN, NY, GA, SC, and MI were allocated in Group 2 and unable to resolve by geography. The separation of NC isolates from TN, NY, GA, SC, and MI in the STRUCTURE analysis supports the clade identified in the genetic distance tree and the clustering observed in the DAPC analysis (Figs 2 and 3). None of the population structure analyses (Provesti’s distance tree, DAPC, or Structure) clustered the 50 *P. capsici* isolates by host or mating type.

**Table 4:** Genetic differentiation of populations of *Phytophthora capsici* from 6 states.

State	F_ST_ values ^a^					
	GA	SC	NC	NY	TN	MI
GA	0.000					
SC	0.026	0.000				
NC	0.114*	0.161*	0.000			
NY	0.000	0.101*	0.166*	0.000		
TN	0.000	0.088	0.126*	0.083	0.000	
MI	0.070	0.213*	0.324*	0.255*	0.208	0.000

^a^F_ST_ values were calculated in GenALEx 6.5. Values followed by * are significantly different from zero at *P* < 0.005, using 999 permutation.

## Discussion

We exploited the *P. capsici* transcriptome to successfully identify 1,855 microsatellite loci using an *in silico* approach. The number of microsatellites detected in *P. capsici* is larger than those discovered in *P. plurivora*, *P. multivora*, *P. pini*, *P. citricola*, or *P. alni* using the 454-pyrosequencing-based method^32,50,51^. Our *P. ramorum* and *P. sojae* transcriptome survey and a *P. nicotiana* whole genome survey yielded higher numbers of microsatellites compared to what the *P. capsici* transcriptome yielded^28^. Relative abundance and relative density of microsatellites in *P. capsici* differs greatly to the observed values for *P. ramorum* and *P. sojae*; we hypothesize that such differences may be explained by the differences among genome sequencing approaches taken for these three species^38,52^. In fact, our results suggest high similarity among the frequencies, relative abundances, and densities of the same repeat unit across the three species. In the different transcriptome sequences analyzed here, trinucleotide microsatellites were the most common motif length, consistent with studies in other *Phytophthora spp*.^28,41^, fungal plant pathogens^43,47,53^, and plants^54^. The same studies also report lower abundance of di-, tetra-, and pentanucleiotides. Purifying selection for motifs that preserve reading frames explains the predominance of trinucleotide microsatellites in coding regions^55^.

We designed primer pairs for more than 80% of the identified microsatellite loci. Our results reveal information about the reliability and quality of the genome assembly. Garnica, *et al*.^41^ used transcriptome sequences of *P. ramorum* and *P. sojae* and had similar primer design success rate, greater than 80%, which is comparable with our findings. Insufficient flanking sequence adjacent to the microsatellite, such as a microsatellite close to the end of a contig, may explain the 20% primer design failure^48^. Gel electrophoresis screening of a set of 50 microsatellite primers in seven *P. capsici* isolates yielded 17 highly polymorphic microsatellite markers. We observed higher amplification efficiency (96.00%) than reported by Wang and Chilvers^53^ (85.00%) and Gagnon, *et al*.^49^ (79.03%) who examined whole genome sequences of *F. virguliforme* and *S. endobioticum* respectively. Amplification efficiency in our microsatellite primer screening might be attributed to the stability of coding regions with low selection pressure^56^. We identified a high number of polymorphic bands from trinucleotide microsatellites. Di-, tetra-, penta- and hexa-nucleotide microsatellites yielded the lowest number of polymorphic bands. The relatively low polymorphism implies that these repeat types are highly conserved across isolates used in this study in contrast with trinucleotides repeats, which may experience mutation and selection pressure for specific amino acids^43^. We further narrowed the list of microsatellites from 17 to 12 based on consistent amplification and polymorphism observed via gel electrophoresis, and confirmed the polymorphism via fragment length analysis.

Polymorphism indexes such as Nei’s expected heterozygosity (0.55) and evenness (0.83) indicated high potential for these markers to describe diversity in the panel of 50 *P. capsici* isolates. Our Nei’s expected heterozygosity values are similar to the ones reported by Gagnon, *et al*.^49^ and Wallace and Quesada-Ocampo^57^ when they characterized microsatellites for *S. endobioticum* and *Pseudoperonospora cubensis* respectively. In a recent study, Gagnon, *et al*.^58^ used microsatellites mined from whole genome sequence data of *P. ramorum* isolates to assess genetic diversity. The markers developed in their study had an average evenness of 0.943 and were able to detect temporal and spatial distribution of *P. ramorum* in Canadian nurseries. In our study, the MLG curve reached a plateau with 11 microsatellite markers out of 12, implying that these markers are sufficient to depict all 50 multilocus genotypes. The ${\bar{r}}_{d}$ calculated for all isolates after clone correction suggested non-recombination, which is probably due to the fact the our sampling contained isolates from different states and fields in the US. *P. capsici* populations are known to stratify by geography since long distance movement is unlikely^12^. The Shannon-Wiener’s index confirmed the presence of high genetic diversity in our sample of 50 *P. capsici* isolates. NC isolates displayed more genetic diversity than *P. capsici* populations reported from 12 different sites in four regions of New York State^16^. Sampling for NC isolates encompassed 7 different sites distributed in three regions of North Carolina. Our results for diversity measurements suggest that the 12 polymorphic microsatellites produced enough resolution for genotyping genetically distinct individuals.

As a first measure to detect population structure, we created a distance-based tree using Provesti’s distance^59^. The genetic distance tree detected some population structure based on geographic regions enabling us to differentiate isolates into two major clusters with 100% bootstrap support. One of the major clusters consisted of isolates mainly from NC while the other included isolates from all other states evaluated here. Such genetic differentiation can be explained by the fact that *P. capsici* exists in geographically isolated subpopulations in the US^12,16^. Our genetic distance tree was unable to separate isolates from SC, NY, TN, GA, and MI, therefore we performed a discriminatory analysis of principal components in order to infer the number of genetic clusters. Interestingly, the clustering of isolates based on the DAPC supported the hypothesis of the genetic distance tree. NC isolates collected across the state consistently formed a separate group relative to the other isolates in the DAPC. Unlike the genetic distance tree the relative position of SC isolates in the DAPC scatter plot suggests underlying population structure between SC and TN, NY, GA, and MI isolates. The clustering observed in the DAPC for NC and SC isolates showed significant genetic differentiation with F_ST_ values larger than 0.05 and in some cases larger than 0.25. Population differentiation statistics and the AMOVA analysis supported our hypothesis that the 12 microsatellite markers developed here are able to separate *P. capsici* isolates by geographic origin. Even though the AMOVA by host is significant, it only represents 6.38% of the variation between isolates. Two major clusters were detected in the STRUCTURE analysis using the admixture model. We observed some resemblance between the two major groups in the genetic distance tree and the two clusters revealed by STRUCTURE. All NC isolates clustered together in group 1 within the STRUCTURE analysis, supporting our hypothesis that the 12 microsatellite markers are robust and can differentiate isolates by state. We attribute the unresolved group 2 in the STRUCTURE analysis to the low number of isolates per field from SC, NY, TN, GA, and MI. Successful management of *P. capsici* requires a detailed knowledge of regional pathogen population structure and distribution^3^. Conducting surveys of local *P. capsici* populations and examining how they stratify can direct the deployment of management strategies in a more efficient way. The 12 microsatellite markers discovered here represent powerful and inexpensive tools for describing the population structure of *P. capsici* at a regional scale.

*P. capsici* is considered a pathogen with high evolutionary potential, because sexual reproduction increases the probability of developing genotypes that can overcome host resistance or are resistant to fungicides^9^. These novel genotypes could be leading to new epidemics that become isolated due to the soilborne dispersal mechanism of the pathogen^3^, and therefore understanding the phenotypic variation and genotypic structure in a particular region becomes highly desirable. Monitoring the genetic structure of the pathogen along with phenotypic monitoring of fungicide resistance, mating type, and virulence, allow for strategizing management. For example, if a genetic cluster was found to be more virulent to a particular host, it would be valuable to know the geographic location of isolates belonging to that genetic cluster so that one avoids planting a susceptible crop in that area^60^. Microsatellites as described by Cooke and Lees^23^ represent a powerful molecular marker for genetic analysis; they are affordable and reproducible among research groups. Even though genomic approaches are available now such as genotyping by sequencing, the *P. infestans* community still relies on microsatellite markers to understand the pathogen biology and its population dynamics^61–63^. In fact, Dunn, *et al*.^16^ emphasized the need for additional polymorphic markers to increase resolution and detect greater diversity in populations of *P. capsici*. Our study delivers a set of highly polymorphic microsatellite markers to the *P. capsici* community: these 12 microsatellites represent valuable tools to understand the structure and diversity of *P. capsici* populations, allowing for comparisons of datasets generated by different research groups. We foresee an additional application for our microsatellites as transferable markers across species complexes. Quesada-Ocampo, *et al*.^12^ used SNPs from several genomic regions to separate *P. capsici* and *P. tropicalis* in a STRUCTURE analysis, however, their SNP markers were unable to resolve an intermediate cluster containing both species. Microsatellites developed from coding regions are highly transferable to closely related species^43,64,65^. Since *P. capsici* is part of a species complex, we hypothesize that our microsatellites can distinguish among closely related species such as *P. tropicalis* and *P. glovera*. Further experiments should be completed to validate this hypothesis.

Taken together, our findings demonstrate that 12 microsatellites developed from the transcriptome sequences of *P. capsici* are highly polymorphic in a diverse panel of isolates, describe diversity of local populations, and may be transferable to species closely related to *P. capsici*. These microsatellites can be used to analyze the genetic structure of particular geographic regions and determine genotypes present in fields. The information generated by population studies using these markers may allow for better deployment of resistant cultivars if a correspondence between genetic cluster and virulence is found for a particular host. We are interested in using these markers to describe the until now unknown population structure of *P. capsici* in North Carolina and combining that knowledge with phenotypic information regarding fungicide resistance, mating type, and virulence.

## Materials and Methods

### Identification and analysis of microsatellites in the predicted transcriptomes of *P. capsici*, *P. sojae*, and *P. ramorum*

Predicted transcriptomes from genome sequences of isolates LT1534 of *P. capsici*^38^, Pr-102 of *P. ramorum*^52^, and P6497 of *P. sojae*^52^ were downloaded as FASTA files from publically available databases at http://genome.jgi.doe.gov/Phyca11/Phyca11.home.html, http://genome.jgi.doe.gov/ramorum1/ramorum1.home.html, and http://genome.jgi.doe.gov/Physo3/Physo3.home.html. The transcriptome data was searched for microsatellite motifs of one to six nucleotides in length using the program MISA (MIcroSAtellite identification tool)^39^. Search criteria were set for identification of at least 10 repeat units for mono-nucleotides, six for di-nucleotides and five for tri-, tetra-, penta-, or hexa-nucleotides. Results were compared among species for abundance, frequency, distribution, and relative density of each repeat unit in the transcriptomes as described previously^46^.

### Microsatellite marker development

The MISA output files and Perl programming were used to extract the coordinates of identified microsatellites in the *P. capsici* transcriptome and generate input files for designing primers with the Primer3 program^66^. Primer3 identified primer pairs flanking each microsatellite loci with a melting temperature between 57 °C and 63 °C with an optimum at 60 °C, GC content between 20 and 80% with an optimum of 50%, and PCR products with expected length between 100 and 300 bp. The M13 sequence (5′-GACGGCCAGT-3′) was added at the 5′ end of each forward primer in order to allow for later fluorescent product labeling and fragment analysis as previously described^67^.

### Isolates, culture conditions, and DNA extraction

A panel of seven single-spore *P. capsici* isolates were selected for primer validation via gel electrophoresis (Supplementary Table S1). This panel included one isolate from North Carolina (NC19385); one from New Jersey (R328); two from South Carolina (RCZ-11, WLB-8); two from Michigan (12889, SP98); and the isolate used in the *P. capsici* genome sequencing project (LT1534). All isolates were transferred from long term storage cultures to V8 juice broth as described previously^12,68^. Mycelia were harvested through filtration, immediately lyophilized, and stored at −80 °C.

Genomic DNA was isolated using the following phenol-chloroform protocol: for each isolate, 200 mg of lyophilized mycelium were ground on an OMNI Bead Ruptor 24 (Omni International, Kennesaw, GA) at a speed of 5 m/s for 60 s, adding 2 mm glass beads. Subsequently, 600 µl of extraction buffer (0.5 M Tris pH 8.0, 5 M NaCl, 0.5 M EDTA, 10% SDS) was added to the powdered tissue and homogenized by vortexing. Samples were treated with 4 µl of RNAase A (Invitrogen Life Technologies, Grand Island, NY) and incubated for 10 min at room temperature with occasional gentle mixing. 500 µl of phenol was added, and the mixture was centrifuged for 10 min at 21,130 g. The supernatant was transferred to a fresh tube and an equal volume of Phenol:Chloroform was added and mixed by gentle inversion; the mixture was centrifuged for 10 min at 21,130 *g*. The aqueous supernatant was transferred to a new tube and 0.8 volume of Isopropanol was added. Tubes were incubated at −20 °C overnight. After incubation, all samples were centrifuged for 15 min at 21,130 g, the supernatant was discarded and the pellet was precipitated with 80% Ethanol. The pellet was dissolved in 200 µl of sterile water. Quality and integrity of genomic DNA were estimated by measuring the 260/280 nm ratio in a NanoDrop ND-1000 spectrophotometer (Thermo Scientific, Wilmington, DE) and by electrophoresis in a 1% agarose gel in 0.5 × Tris-borate-EDTA buffer stained with ethidium bromide (5 µg/ml) for visualization.

### Microsatellite evaluation

Ten microsatellite primer pairs each from di-, tri-, tetra-, penta-, and hexa-nucleotide motif length were randomly selected from the Primer3 output to detect polymorphism among 7 *P. capsici* isolates (Supplementary Table S2). Microsatellite primers selected were synthesized by Integrated DNA Technologies (Coralville, IA). Polymerase chain reactions (PCR) were executed in a T100 thermocycler (Bio-Rad, Hercules, CA). 20 µl of reaction volume contained 1 µl of genomic DNA at 50 ng/µl, 10 µl of GoTaq® Green Master Mix (2X GoTaq Green Master consisting of GoTaq Green Reaction Buffer, 400 µM of each dNTP, and 3 mM MgCl_2_; Promega, Madison, WI), 1 µl of each 10 µM primer, and 7 µl of sterile water. PCR cycling conditions included an initial denaturation at 94 °C for 3 min; followed by 35 cycles of denaturation at 94 °C for 30 s, annealing at 53 °C for 30 s and extension at 72 °C for 30 s; with a final extension at 72 °C for 5 min. PCR products were analyzed for polymorphism by electrophoresis in 4% agarose gels and visualized by ethidium bromide staining. A 100 bp DNA ladder (Invitrogen Life Technologies, Grand Island, NY) was used to estimate allele size.

### Population analyses using the microsatellite markers

To determine the effectiveness of the microsatellite markers developed in this study for understanding population genetic structure and diversity, a subset of 12 microsatellite primers were selected on the basis of polymorphisms in agarose gels. These microsatellite markers were further analyzed across 50 *P. capsici* isolates from different states, hosts, and mating types via fragment analysis (Table 1). PCR products of the polymorphic microsatellite primers were subjected to a second round of PCR. In these PCRs the forward primer of each microsatellite was 5′ labelled with four fluorescent dyes (6-FAM, VIC, NED, and PET from Applied Biosystems, Foster City, CA). Prior to fragment analysis, 1 µl of PCR product from 4 microsatellite loci labeled with different fluorescent dyes were pooled together and diluted in ratio 1:100 with sterile dH_2_O. In order to accurately detect variability in microsatellite length among isolates, a genotyping reaction was performed suspending 1 µl of diluted PCR product in 8.5 µl HiDi Formamide and 0.5 µl of Gene Scan 600LIZ dye Size Standard (Applied Biosystems, Foster City, CA). Genotyping reactions were subsequently performed by the NCSU Genomic Science Laboratory (GSL, Raleigh, NC) on an ABI 3730xl DNA Analyzer (Applied Biosystems, Foster City, CA).

Allele sizes were called using the Microsatellite Plugin in Geneious version R9.0.5 (Biomatters, New Zealand). In order to reduce the risk of genotyping stutter peaks, we manually removed from the analysis peaks lower than 5% of the signal of the tallest peak at any given locus. We assumed two alleles to be present at each loci because *P. capsici* is a diploid organism belonging to the class Oomycota. Descriptive population statistics such as the number of alleles per locus (Na), heterozygosity (He), evenness, and the genotype accumulation curve were estimated using the functions “gac” and “locus_table” from the package *poppr* in RStudio (version 0.99.903 and R version 3.2.4; R Core Team, Austria)^69–71^.

We employed the function “poppr” using the stratified clonecorrected data (by states, host, and mating type) to calculate the number of multilocus genotypes, the Shannon-Wiener index of genetic diversity and other summary statistics. The standardized index of association (${\bar{r}}_{d}$) was calculated with the “ia” function^72^. We built a genetic distance tree to assess genetic relatedness between isolates using Provesti’s distance with 1,000 bootstrap replicates^59^. Because the set of microsatellite markers discovered in this study are intended to elucidate population structure of *P. capsici*, we assessed whether the 50 *P. capsici* isolates significantly structure by state, host, or mating type by implementing the Analysis of Molecular Variance (AMOVA) included in the *poppr* package. To test for differentiation between *P. capsici* isolates from different states, pairwise F_ST_ values were estimated, and significance levels were tested with 999 permutations using GenAlEx 6.5^73^. The presence of an underlining structure was examined using the Discriminant Analysis of Principal Components (DAPC) with the R package *adegenet*^74^, and the Bayesian Markov Chain Monte Carlo (MCMC) clustering model using the software STRUCTURE v2.3.4^75^. The values for length of burnin, chain replication, and lambda were set at 50,000, 250,000, and 1 respectively. Isolates membership was inferred for one to 10 clusters and the optimal K was chosen by computing ΔK using STRUCTURE HARVESTER v.0.6.94^76^. Population structure figure defining the state of origin was obtained using Microsoft Excel (version 15.20).

## Electronic supplementary material

Supplementary Information

Supplementary Table S2

Supplementary Table S4
