# Supplementary material for: Analysis of microsatellites from transcriptome sequences of Phytophthora capsici and applications for population studies
Source: Sci Rep. 2018 Mar 26;8:5194. doi: 10.1038/s41598-018-23438-8 (PMC5980080; doi:10.1038/s41598-018-23438-8)
Supplement: Supplementary file 1 — Supplementary Information [file 41598_2018_23438_MOESM1_ESM.pdf]

Supplementary information

Analysis of microsatellites from transcriptome sequences of *Phytophthora capsici* and applications for population studies

C. H. Parada-Rojas[1] and L. M. Quesada-Ocampo[1*]

[1]Department of Plant Pathology, North Carolina State University, Raleigh, NC 27695, USA.

[*]Corresponding author: L. M. Quesada-Ocampo, E-mail: lmquesad@ncsu.edu

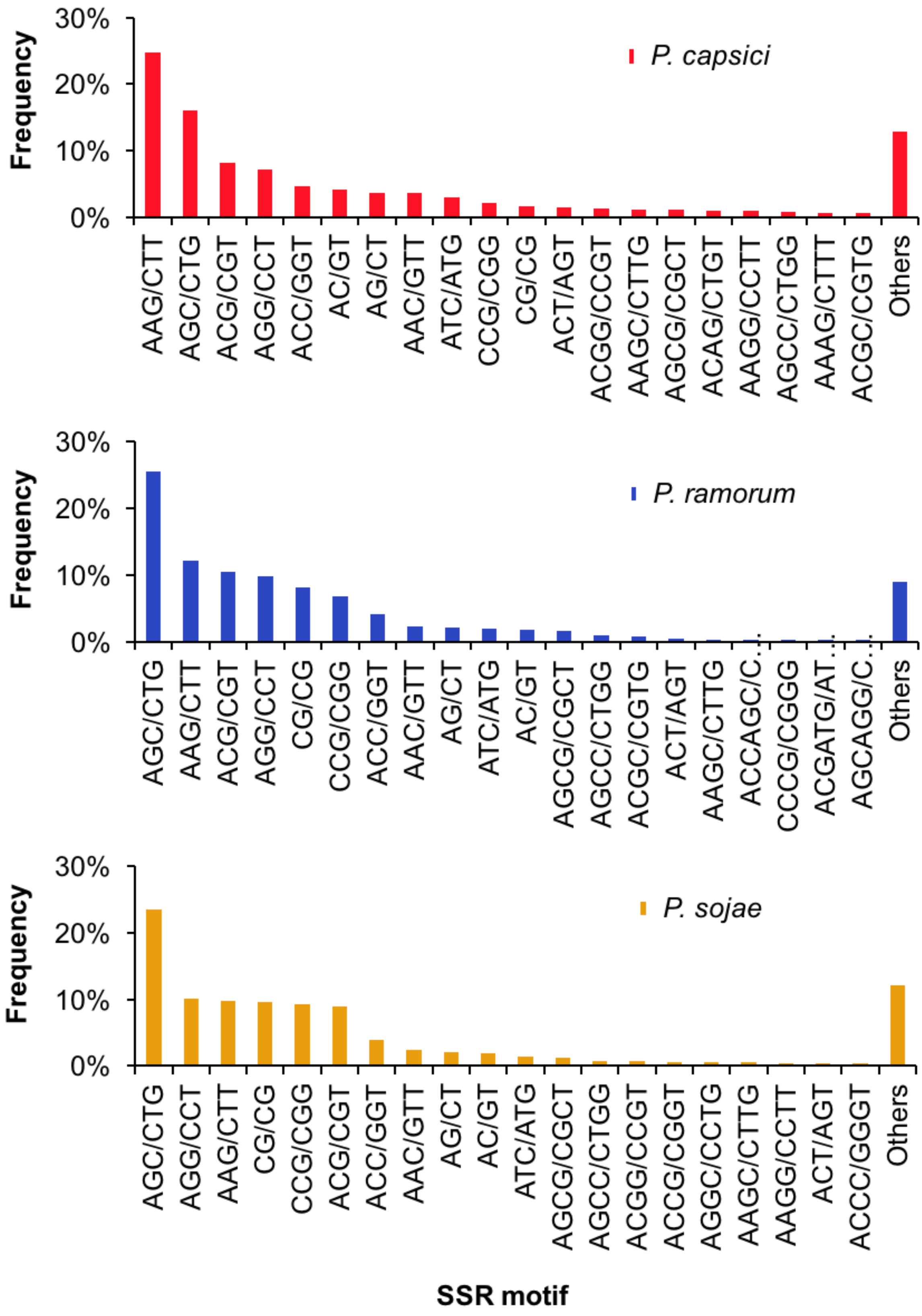

Supplementary Fig. S1. Frequency of the most abundant microsatellite repeat motifs in the three *Phytophthora* species. **(red)** *P. capsici*. **(blue)** *P. ramorum*. **(yellow)** *P. sojae*.

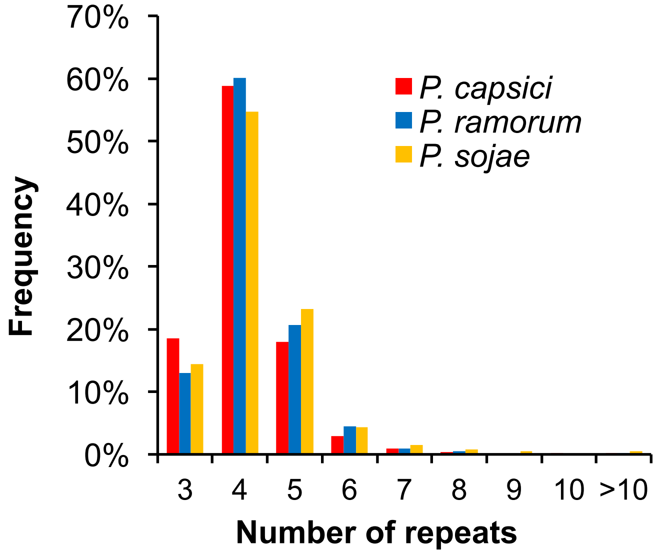

Supplementary Fig. S2. Distribution of microsatellites in *P. capsici, P. ramorum,* and *P. sojae* by number of repeats.

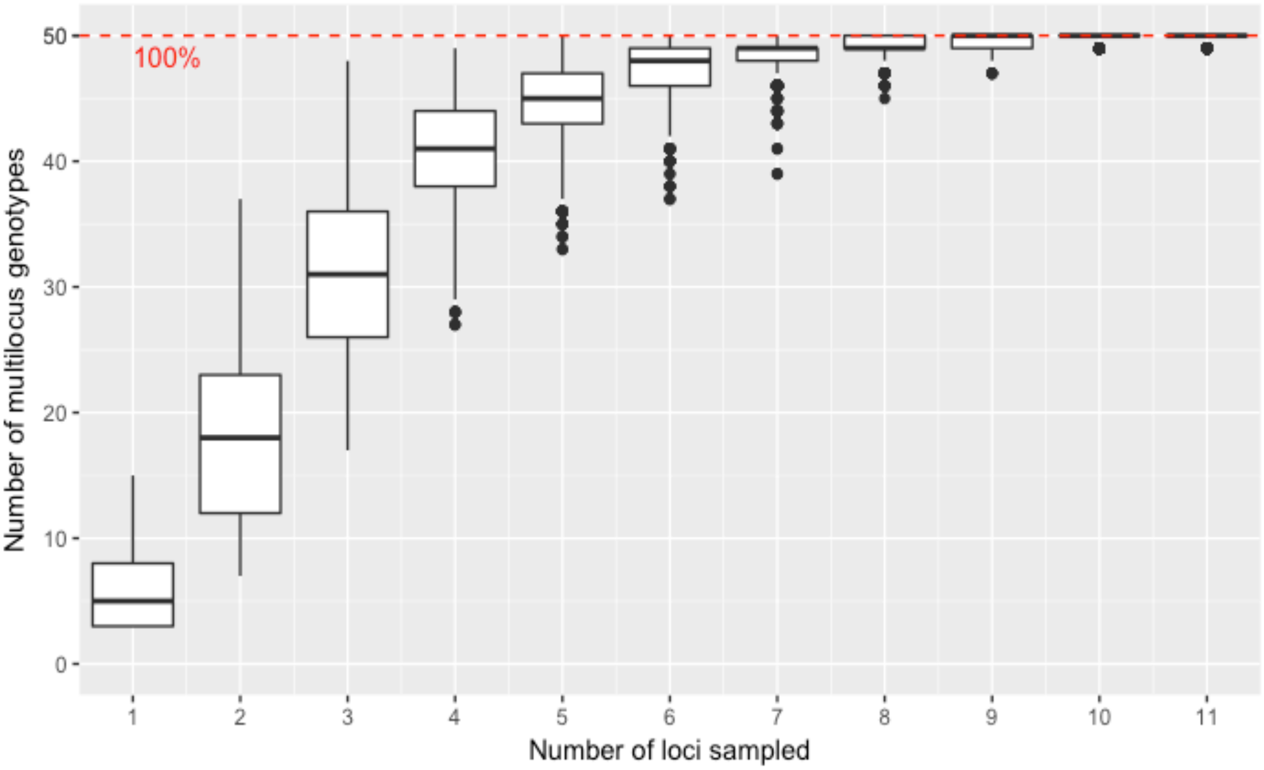

Supplementary Fig. S3. Genotype accumulation curve. 50 multilocus genotypes of *P. capsici* (100%) are detected using 11 microsatellite loci.

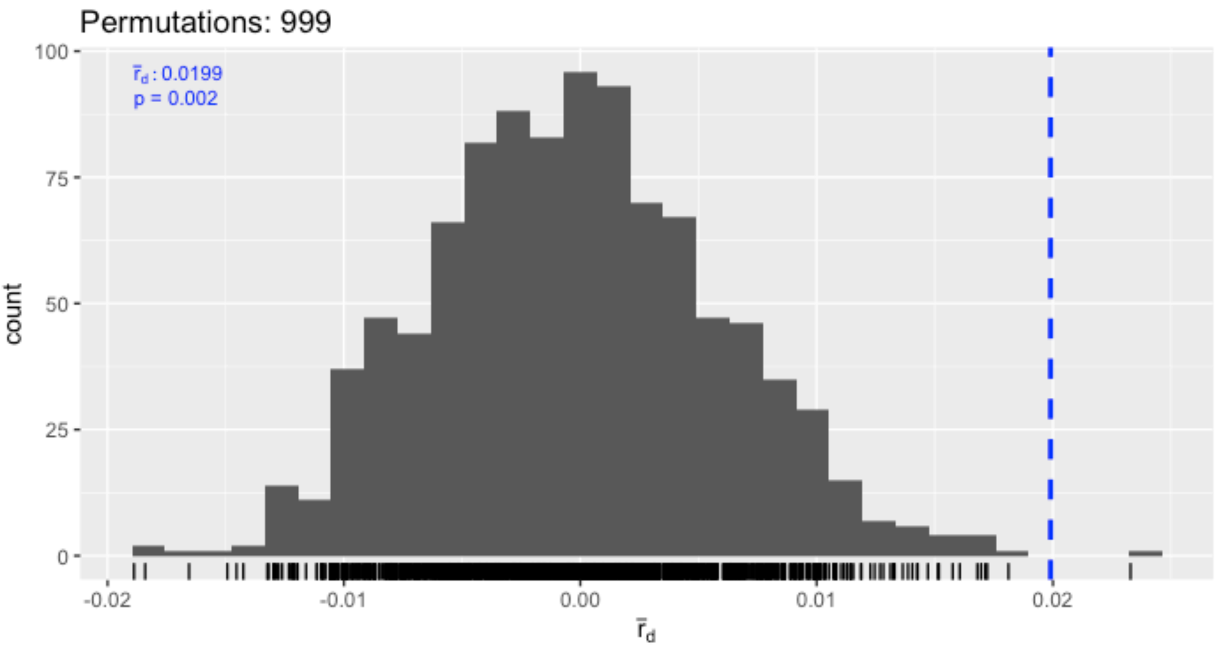

Supplementary Fig. S4. Index of association in *Phytophthora capsici* isolates calculated from 999 permutations. The distribution of I^A for all *P. capsici* isolates showing a rejection of the null hypothesis, thus suggesting a clonal population.

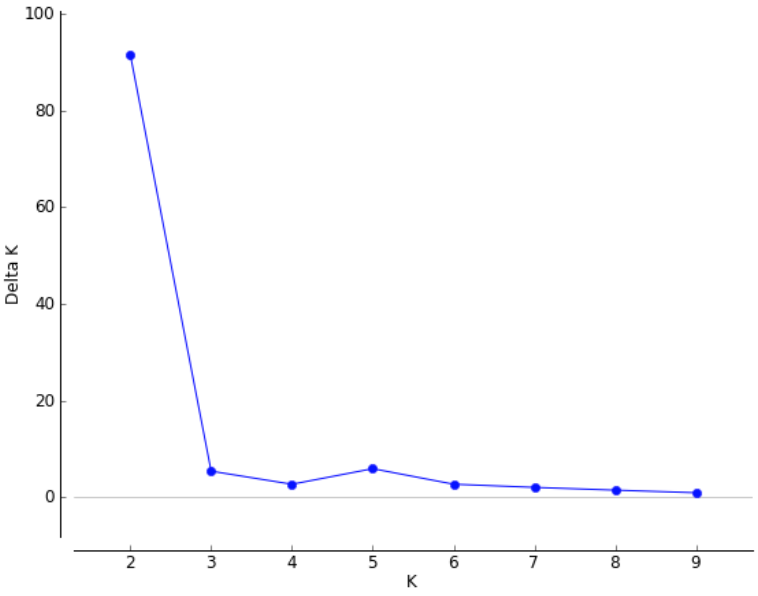

Supplementary Fig. S5. Most optimal number of clusters for 30 *P. capsici* isolates calculated by STRUCTURE HARVESTER. Delta K values plotted against the number of probable clusters (K).

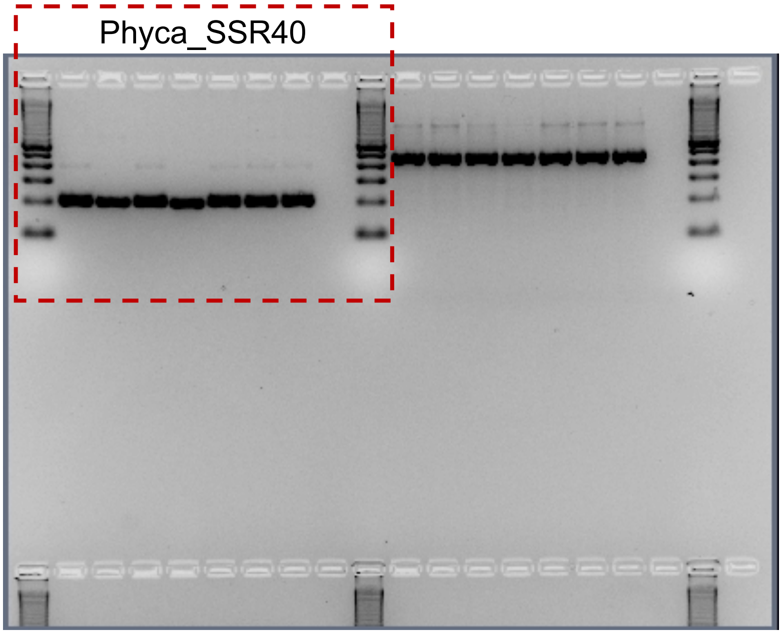

Supplementary Fig. S6. Full-length gel including amplification profile from 7 *P. capsici* isolates using the microsatellite marker Phyca_SSR40. Image was captured using standard settings in GEL DOC UV SYSTEM (Biorad Company, CA, USA).

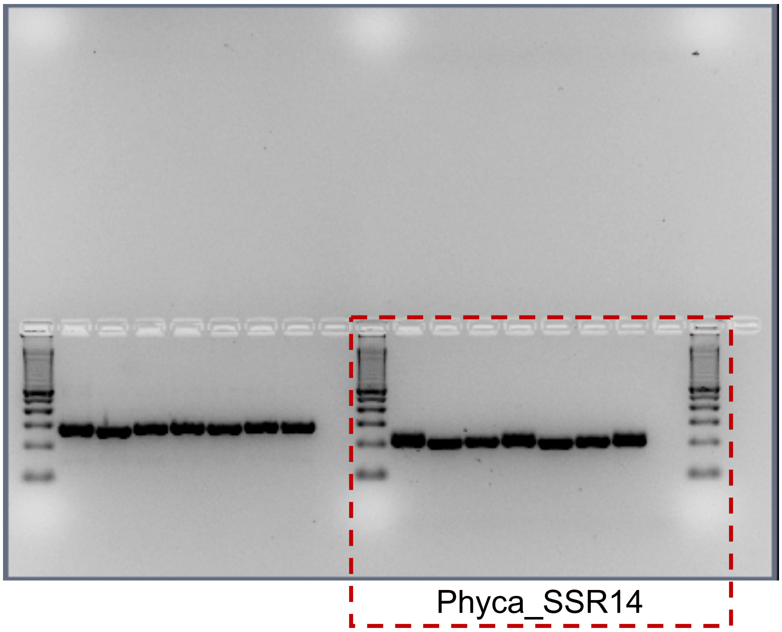

Supplementary Fig. S7. Full-length gel including amplification profile from 7 *P. capsici* isolates using the microsatellite marker Phyca_SSR14. Image was captured using standard settings in GEL DOC UV SYSTEM (Biorad Company, CA, USA).

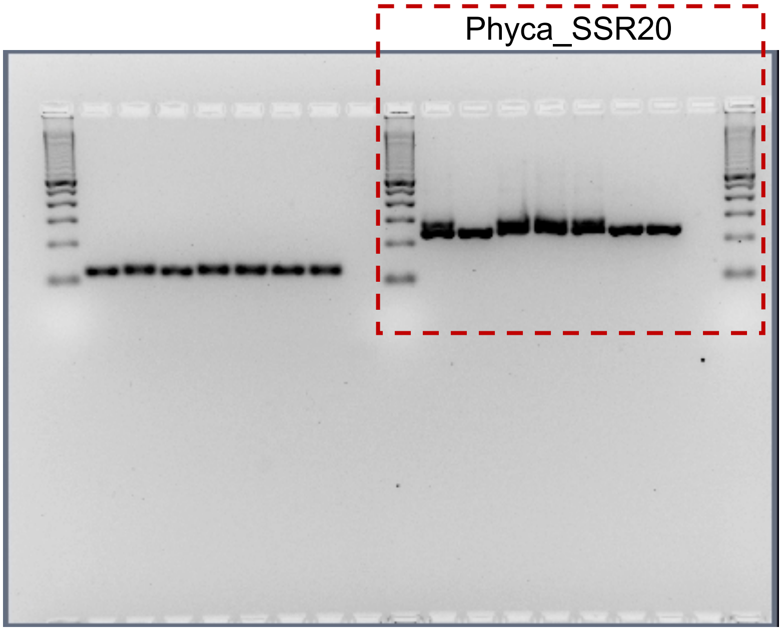

Supplementary Fig. S8. Full-length gel including amplification profile from 7 *P. capsici* isolates using the microsatellite marker Phyca_SSR20. Image was captured using standard settings in GEL DOC UV SYSTEM (Biorad Company, CA, USA).

Supplementary Table S1. *Phytophthora capsici* isolates used for microsatellite evaluation

**Isolate**	**Host**	**Origin**	**Mating type**	**Source**
NC19385	Watermelon	North Carolina	A1	Quesada
R328	Pepper	New Jersey	A2	Ristaino
RCZ-11	Zucchini	South Carolina	A2	Kousik
WLB-8	Watermelon	South Carolina	A1	Kousik
12889	Pepper	Michigan	A1	Hausbeck
SP98	Pumpkin	Michigan	A2	Hausbeck
LT1534	-	Inbred isolate	A2	Lamour

Supplementary Table S2. Details of 50 *P. capsici* microsatellite loci validated for PCR in 7 *P. capsici* isolates. XLXS file

Supplementary Table S3. Number and distribution of microsatellites in *P. capsici*, *P. ramorum,* and *P. sojae* transcriptome.

**Description**	***P. capsici***	***P. ramorum***	***P. sojae***
Total number of sequences examined	19,805	15,743	26,584
Total size of examined sequences (Mb)	20.36	22.49	31.41
Total number of identified microsatellites	1,855	3,278	6,157
Number of microsatellites containing sequences	1,620 (8.17%)	2,620 (16.64%)	4,509 (16.96%)
Number of sequences containing > 1 microsatellite	180 (0.91%)	472 (2.99%)	1,118 (4.21%)
Number of compound microsatellites	75 (4.04%)	180 (5.49%)	502 (8.15%)
Total relative abundance (SSRs/Mb)	91.09	145.75	196.04
Total relative density (bp/Mb)	1280.05	2181.37	3190.98

Supplementary Table S4. Primer pairs designed for the amplification of microsatellite loci in *P. capsici*. XLXS file
